# Supplementary material for: Rapid detection of extended spectrum β-lactamase producing Escherichia coli isolated from fresh pork meat and pig cecum samples using multiplex recombinase polymerase amplification and lateral flow strip analysis
Source: PLoS One. 2021 Mar 15;16(3):e0248536. doi: 10.1371/journal.pone.0248536 (PMC7959403; doi:10.1371/journal.pone.0248536)
Supplement: Attachment — Submitted filename: Response to reviewers.docx [file pone.0248536.s001.docx]

**February 25, 2021**

RE: Rapid detection of extended spectrum β-lactamase producing *Escherichia coli* isolated from fresh pork meat and pig caecum samples using multiplex recombinase polymerase by Sitthichai Kanokudom, Thachaporn Assawakongkarat, Yukihiro Akeda, Panan Ratthawongjirakul, Rungtip Chuanchuen, Nuntaree Chaichanawongsaroj

**Dear Editor-in-Chief,**

We thank reviewers for constructive comments. We have revised our manuscript and the followings are responses to comments of reviewers.

**Response to Editor Comments:**

- Please correct discrepancy in Table 3. It is noted than in Table 3, it is stated that n=140. Column 2 shows that 130 were PCR positive for blaCTX-M and column 3 shows the breakup of different combinations - either blaCTX-M alone or along with blaOXA and blaTEM and the total is 129. It should be 130 as per column 2. Why this discrepancy?

Response: The discrepancy in Table 3 is correct. The total number of *bla*_CTX-M_ detected by PCR was 130, while RPA-LFA demonstrated only 129 of *bla*_CTX-M_. One PCR positive sample of *bla*_CTX-M_ and *bla*_TEM_ gave false negative by RPA-LFA.

The clarification was incorporated in page 15, line 6-8: Of 130 *bla*_CTX-M_ PCR-positive samples, a discrepancy was revealed in one of the *bla*_CTX-M_, *bla*_TEM_ samples by RPA-LFA (Table 3).

Addition correction has been done in Table 3, the number of *bla*_CTM-M_ alone and *bla*_CTX-M_, *bla*_TEM_ detected by RPA-LFA were 47 and 80, respectively.

**Response to Reviewer #1:**

1. I would recommend the manuscript to be edited by native speaker of English prior to re-submission.

Response: The revised manuscript has already English proof by AJE before re-submission. The corrections have been done as in “Manuscript with track changes”.

2. Fig 1. Spelling of “solution” need to be corrected

Response: This typing error “solution” in Fig.1 has already corrected.

3. Pictures are not very clear. Could these photographs be improved at all?

Response: This concern of photographs has already improved and check by Preflight Analysis and Conversion Engine (PACE) digital diagnostic tool to ensure that figures meet PLOS requirements.

Additional correction.

1. The revised manuscript has been rechecked and edited to meets PLOS ONE’s style requirements.
2. The detail of figure legend in Fig 1. has been incorporated in page8, line 17-21: “The C-PAS4 membrane strip has 3 position markers (red lines) and four imprinted tag oligonucleotides from the bottom to top lines (tag1-4; Left). The C-PAS4 membrane strip is inserted into a developing solution containing RPA amplicons (middle) for 5-10 min to allow the RPA amplicons to be trapped by the complementary tag sequences and generate visible blue lines (CTX, SHV, OXA) at the relevant positions, as indicated (right). ”.

Best Regards,

Nuntaree Chaichanawongsaroj, Ph.D.

Department of Transfusion Medicineand Clinical Microbiology, Faculty of Allied Health Sciences, Chulalongkorn University, Bangkok 10330, Thailand. Phone: (662) 218-1084 ext. 324,

Fax: (662) 218-1083,

Email: nuntaree@gmail.com
